# Investigation on nitridation processes of Sr_2_Nb_2_O_7_ and SrNbO_3_ to SrNbO_2_N for photoelectrochemical water splitting

**DOI:** 10.1038/s41598-018-34184-2

**Published:** 2018-10-26

**Authors:** Masanori Kodera, Yosuke Moriya, Masao Katayama, Takashi Hisatomi, Tsutomu Minegishi, Kazunari Domen

**Affiliations:** 1 Department of Chemical System Engineering, School of Engineering, The University of Tokyo, 7-3-1 Hongo, Bunkyo-ku, Tokyo 113-8656 Japan; 2 Center for Energy & Environmental Science, Shinshu University, 4-17-1 Wakasato, Nagano, 380-8553 Japan; 3 Present Address: Global Aqua Innovation Center for Improving Living Standards and Water-sustainability, Shinshu University, 4-17-1 Wakasato, Nagano, 380-8553 Japan

## Abstract

The processes involved in the nitridation of Sr_2_Nb_2_O_7_ and SrNbO_3_ to SrNbO_2_N were assessed by varying the nitridation time, and the related effects on the physical and photoelectrochemical properties of the nitrided products were investigated. In the case of the layered perovskite-type oxide Sr_2_Nb_2_O_7_, the introduction of nitrogen and the extraction of oxygen took place concurrently, leading to lattice shrinkage and a porous structure. In contrast, during nitridation of the perovskite-type oxide SrNbO_3_, nitrogen was initially introduced without any loss of oxygen, which caused phase separation as a result of a lattice expansion and a charge compensation. The photoelectrochemical properties of obtained SrNbO_2_N under simulated sunlight were found to vary with the oxide precursor used and with the nitridation process.

## Introduction

Hydrogen produced from water using sunlight is an attractive candidate as a next-generation clean fuel. For this reason, the photoelectrochemical (PEC) water splitting reaction has been the subject of significant research following the initial report of the Honda-Fujishima effect^1^. In order to utilize sunlight effectively during PEC water splitting, various oxynitrides and oxysulfides capable of absorbing a wide range of visible wavelengths have been developed^2^. At present, Ta-based (oxy)nitrides such as TaON, Ta_3_N_5_, LaMg_1/3_Ta_2/3_O_2_N, and BaTaO_2_N are being intensively investigated both in powder suspensions and PEC systems, and have exhibited relatively high efficiencies^3–6^. In contrast, studies regarding Nb-based materials remain limited, even though such compounds actually have longer absorption edge wavelengths than the analogous Ta-based materials. It is also notable that Nb is potentially preferable to Ta both with regard to the lower cost and ready supply of the former, although the efficiencies reported for Nb-based catalysts remain very low^7,8^.

Among Nb-based oxynitrides, SrNbO_2_N is a potential candidate for photoanodes in PEC cells. SrNbO_2_N has a perovskite-type structure and an absorption edge at approximately 690 nm^9^. It has been reported that SrNbO_2_N modified with a CoPi cocatalyst functions as a photoanode, generating a current density of 1.5 mA cm^−2^ at 1.23 V vs. a reversible hydrogen electrode (RHE)^10^. Typically, SrNbO_2_N is synthesized from the layered perovskite-type compound Sr_2_Nb_2_O_7_ or from amorphous oxide precursors by heating these materials under an ammonia flow. Sr_5_Nb_4_O_15_, Sr_4_Nb_2_O_9_ and SrNb_2_O_6_ are all possible oxide precursors, although the Sr/Nb ratios in these materials are not the ideal value of unity, thus requiring an additional washing step to remove excess Sr or Nb species after nitridation. Recently, Sr_1-*x*_NbO_3_, having a slightly distorted perovskite-type structure, has been reported as a photocatalyst with an absorption edge of 650 nm, and has been found to work as a photoanode^11^, albeit with very limited PEC performance. Very recently, our own group determined that ANbO_2_N compounds (A = Ba, Sr) synthesized from ANbO_3_ (A = Ba, Sr) function as photoanodes in conjunction with a loaded cocatalyst^12^. As an example, SrNbO_2_N synthesized from SrNbO_3_ has exhibited a current density of 0.4 mA cm^−2^ at 1.2 V vs. RHE under simulated sunlight irradiation. The perovskite-type compound SrNbO_3_ can therefore be used as the oxide precursor for the synthesis of this catalyst.

One of the key factors associated with improving Nb-based oxynitride photocatalysts is control over the nitridation conditions. Although the nitridation conditions for the oxynitrides have already been largely optimized in each material, our understanding of the nitridation mechanism and the relationship between the nitridation processes and PEC properties remains very limited. In the case of Sr_2_Nb_2_O_7_, the overall nitridation reaction can be summarized according to the theoretical equation:

1 $${{\rm{Sr}}}_{2}{{\rm{Nb}}}_{2}{{\rm{O}}}_{7}+2{{\rm{NH}}}_{3}\to 2{{\rm{SrNbO}}}_{2}{\rm{N}}+3{{\rm{H}}}_{2}{\rm{O}}$$

It is considered that the active species during nitridation is NH, NH_2_ or N radical species^13^. In this process, at the same time, the decomposition of ammonia partly produces hydrogen as:

2 $$2{{\rm{NH}}}_{3}\to {{\rm{N}}}_{2}+3{{\rm{H}}}_{2}$$

meaning that a reductive atmosphere is present during nitridation^14^. Nitridation typically changes the crystal structure of the oxide due to the exchange of O^2−^ with N^3−^. As an example, Sr_2_Nb_2_O_7_, initially having a layered perovskite structure, transitions to the perovskite-type material SrNbO_2_N. On the other hand, SrNbO_3_ is believed to undergo nitridation to SrNbO_2_N via the reaction:

3 $${{\rm{SrNbO}}}_{3}+{{\rm{NH}}}_{3}\to {{\rm{SrNbO}}}_{2}{\rm{N}}+{{\rm{H}}}_{2}{\rm{O}}+1/2{{\rm{H}}}_{2}$$

In this case, both the oxide precursor and the resulting oxynitride have a perovskite-type structure. Another feature of this process is that Nb^4+^ is evidently oxidized to Nb^5+^ in spite of the reductive atmosphere during the nitridation. Although the total nitridation reactions from Sr_2_Nb_2_O_7_ or SrNbO_3_ to SrNbO_2_N apparently seems to be relatively simple, as summarized in Eqs (1) and (3), the details of these nitridation processes are not yet known.

In the current work, nitridation of Sr_2_Nb_2_O_7_ and SrNbO_3_ were investigated for various reaction times. The results demonstrate that the nitridation mechanisms for these two oxide precursors are greatly different, resulting in different optical, morphological and PEC properties for the end products.

## Experimental

### Preparation of SrNbO_2_N

Sr_2_Nb_2_O_7_ was synthesized using a flux method^10^. SrCO_3_, Nb_2_O_5_, and RbCl (as a flux) were mixed in a molar ratio of Sr:Nb:Rb = 1:1:5 and then heated in an alumina crucible for 5 h at 1423 K in air. The mixture was subsequently cooled to 1073 K at a rate of 1 K min^−1^ and then allowed to cool to room temperature, followed by washing using a copious amount of distilled water.

To obtain SrNbO_3_, Sr_5_Nb_4_O_15_ was first synthesized by calcining a mixture of SrCO_3_ and Nb_2_O_5_ (at a Sr:Nb molar ratio of 5:4) at 1423 K for 48 h in air. The resulting Sr_5_Nb_4_O_15_ particles were mixed with Nb metal powder at a Sr_5_Nb_4_O_15_:Nb molar ratio of 1:1 in an agate mortar, and then calcined under an Ar flow of 100 mL min^−1^ at 1773 K for 20 h to synthesize SrNbO_3_^11,15^. During this calcination, the sample was wrapped in a 5 cm square sheet of Nb foil and placed on an alumina boat.

In a typical nitridation, approximately 0.5 g of the oxide precursor was placed on an alumina boat, heated at 10 K min^−1^ to 1173 K and then held for 1, 5, 10, 20, 30, or 40 h at 1173 K under a 250 mL min^−1^ NH_3_ flow. Finally, samples were allowed to cool naturally to room temperature.

### Preparation of photoelectrodes

Photoelectrodes composed of SrNbO_2_N were prepared by a particle transfer method^16^. The photocatalyst powder was first suspended in 2-propanol and then dropped onto a glass plate, after which the solution was dried at room temperature. A layer of Nb (approximately 300 nm thick) was then deposited on the dried particles using radio-frequency (RF) magnetron sputtering, followed by the sputtering of a thick Ti layer (>5 μm). The resulting film incorporating the photocatalyst powder was transferred to another glass plate using double sided carbon tape and ultrasonicated in distilled water to remove particles that were not firmly held by the metal film.

### Photoelectrochemical measurements

Current-potential curves for the SrNbO_2_N photoanodes were acquired using a typical three-electrode configuration under intermittent illumination with simulated sunlight provided by a Xe lamp equipped with an air mass (AM) 1.5G filter (San’ei Denki, XES-40S2-CE). Ag/AgCl in a saturated KCl solution and Pt wire were employed as the reference and counter electrodes, respectively. The electrolyte was a 0.2 M aqueous sodium phosphate solution adjusted to a pH of 13 by NaOH addition. In all cases, a CoPi cocatalyst was deposited by electrodeposition at 1.7 V vs. RHE for 200 s^17^.

### Characterization

Samples were characterized using X-ray diffraction (XRD; Rigaku, RINT-Ultima III), UV-vis diffuse reflectance spectroscopy (DRS; JASCO, V-670DS), field-emission scanning electron microscopy (FE-SEM; Hitachi, S-4700, and JEOL, JSM-7001F) and field-emission transmission electron microscopy (FE-TEM; JEOL, JEM-2800). The DRS reflectance (*R*) data were converted to the Kubelka-Munk function using the equation f(*R*) = (1-*R*)^2^/(2 *R*). The relative weight (*W*_*R*_) of each specimen was determined from the equation *W*_*R*_ = (*W*_*a*_−*W*_0_)/(*W*_*b*_−*W*_0_), where *W*_*a*_, *W*_*b*_, and *W*_0_ are the total weight of the powder and the alumina boat after nitridation, the total weight of the powder and alumina boat before nitridation, and the weight of the alumina boat, respectively. Elemental analysis was performed using inductively coupled plasma atomic emission spectroscopy (ICP-AES; Shimadzu, ICPS-8100) and oxygen/nitrogen combustion analysis (ON analysis; Horiba, EMGA-620W/C). Nitrogen adsorption isotherms for the samples were acquired at 77 K with a BELSORP-mini instrument (MicrotracBEL). The relative surface areas of the samples were determined using the Brunauer-Emmett-Teller (BET) model.

## Results and Discussion

### Structural analysis

**Figure 1:**
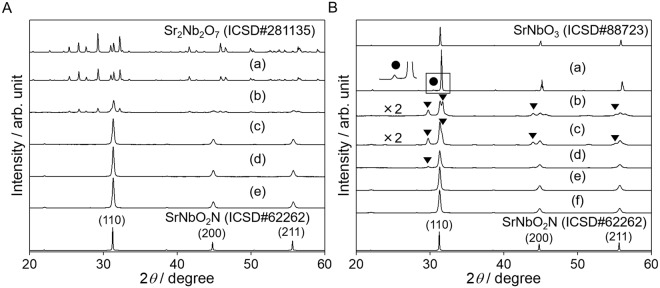
XRD patterns for SrNbO_2_N obtained from nitridation of (**A**) Sr_2_Nb_2_O_7_, and (**B**) SrNbO_3_. Legend: (a) oxide precursor and (b–f) nitrided samples obtained after nitridation for (b) 1, (c) 5, (d) 10, (e) 20, and (f) 30 h. Closed triangles (▼) and closed circles (●) indicate Sr_5_Nb_4_O_15_ and Sr_7_Nb_6_O_21_, respectively. The inset in (**B**) is a magnification of the region indicated by the box.

The Sr_2_Nb_2_O_7_ oxide precursor was synthesized by a flux method and then subsequently nitrided for various nitridation time to investigate the process that generates SrNbO_2_N. Figure 1A shows the XRD patterns obtained from the Sr_2_Nb_2_O_7_ oxide precursor and the nitridation product SrNbO_2_N. It is evident that Sr_2_Nb_2_O_7_ with a layered perovskite-type structure was obtained without any impurity phases. As the nitridation time was increased, the diffraction peaks due to the oxide precursor became less intense, while peaks attributed to a perovskite-type SrNbO_2_N phase appeared. After 10 h, peaks from the oxide precursor completely disappeared. As shown in Table S1, the full width at half maximum (FWHM) values of the (110) diffraction peaks generated by the SrNbO_2_N did not change after 5 h. This result indicates that the crystallinity was not improved by prolonging the nitridation for more than 5 h.

**Figure 2:**
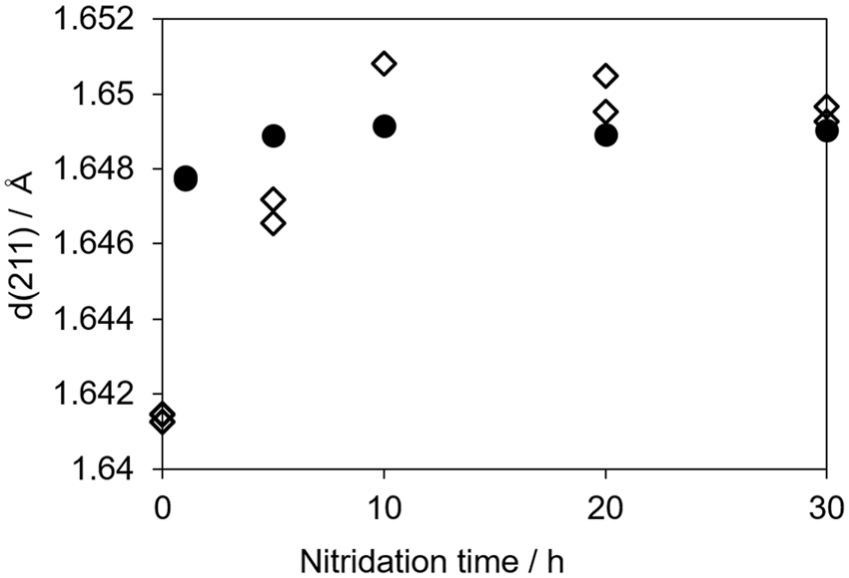
d-values of (211) peaks for SrNbO_2_N nitrided from different oxide precursors. Closed circles and open diamonds indicate Sr_2_Nb_2_O_7_ and SrNbO_3_ series, respectively.

Figure 1B presents XRD patterns for the SrNbO_3_ oxide precursor and the nitridation product SrNbO_2_N. SrNbO_3_ was synthesized by a solid state reaction under an Ar flow, and a small amount of a Sr_7_Nb_6_O_21_ phase was evidently present as an impurity phase. It has been reported that stoichiometric SrNbO_3_ is not readily obtained, while Sr-poor compositions having the formula Sr_1-*x*_NbO_3_ are relatively stable^11^. Moreover, ICP-AES data demonstrated that the Sr/Nb ratio in the oxide precursor was 0.96 (Table S2), most likely due to the vaporization of Sr species during calcination at 1773 K. Therefore, it is probable that the synthesized oxide was a mixture of Sr_1-*x*_NbO_3_ and a small amount of Sr_7_Nb_6_O_21_. Since SrNbO_3_ and SrNbO_2_N both have a perovskite-type structure and their lattice constants are very similar (the lattice mismatch is only 0.7%), it was anticipated that the nitridation would proceed without any change in the crystal structure. Surprisingly, it was found that, following the initial stage of nitridation, a Sr_5_Nb_4_O_15_ phase appeared and then gradually disappeared as SrNbO_2_N was formed. In addition, the Sr_7_Nb_6_O_21_ phase disappeared after 1 h of nitridation. As shown in Fig. S1, the diffraction peaks attributed to a perovskite-type structure were shifted to lower angles as the nitridation progressed. It is notable that the diffraction peaks associated with SrNbO_3_ and SrNbO_2_N could not be separated, indicating that a solid solution of SrNbO_3_ and SrNbO_2_N was partly formed in this stage. Figure 2 indicates d-values of (211) diffraction peaks. In the case of SrNbO_2_N nitrided from Sr_2_Nb_2_O_7_, d-values became constant after nitridation for 5 h. On the other hand, in the case of SrNbO_3_ series, d-values first became larger then gradually reduced after 10 h. It is expected that the introduction of nitrogen increase the lattice constant since the ionic radius of N^3−^ is larger than that of O^2−^. Another possibility is the introduction of oxygen vacancies which were produced during nitridation. This effect will be discussed in detail later. The FWHM values for the (110) diffraction peaks resulting from a perovskite structure initially increased and then decreased with increasing nitridation time, although these FWHM values remained larger than those from Sr_2_Nb_2_O_7_ as shown in Table S1. These data demonstrate that the SrNbO_2_N synthesized from SrNbO_3_ had a lower degree of crystallinity than that obtained from Sr_2_Nb_2_O_7_. Consequently, nitridation of the perovskite-type material SrNbO_3_ to the perovskite-type product SrNbO_2_N proceeded to some extent, but with a phase separation during the initial stage of nitridation.

### Morphological properties

**Figure 3:**
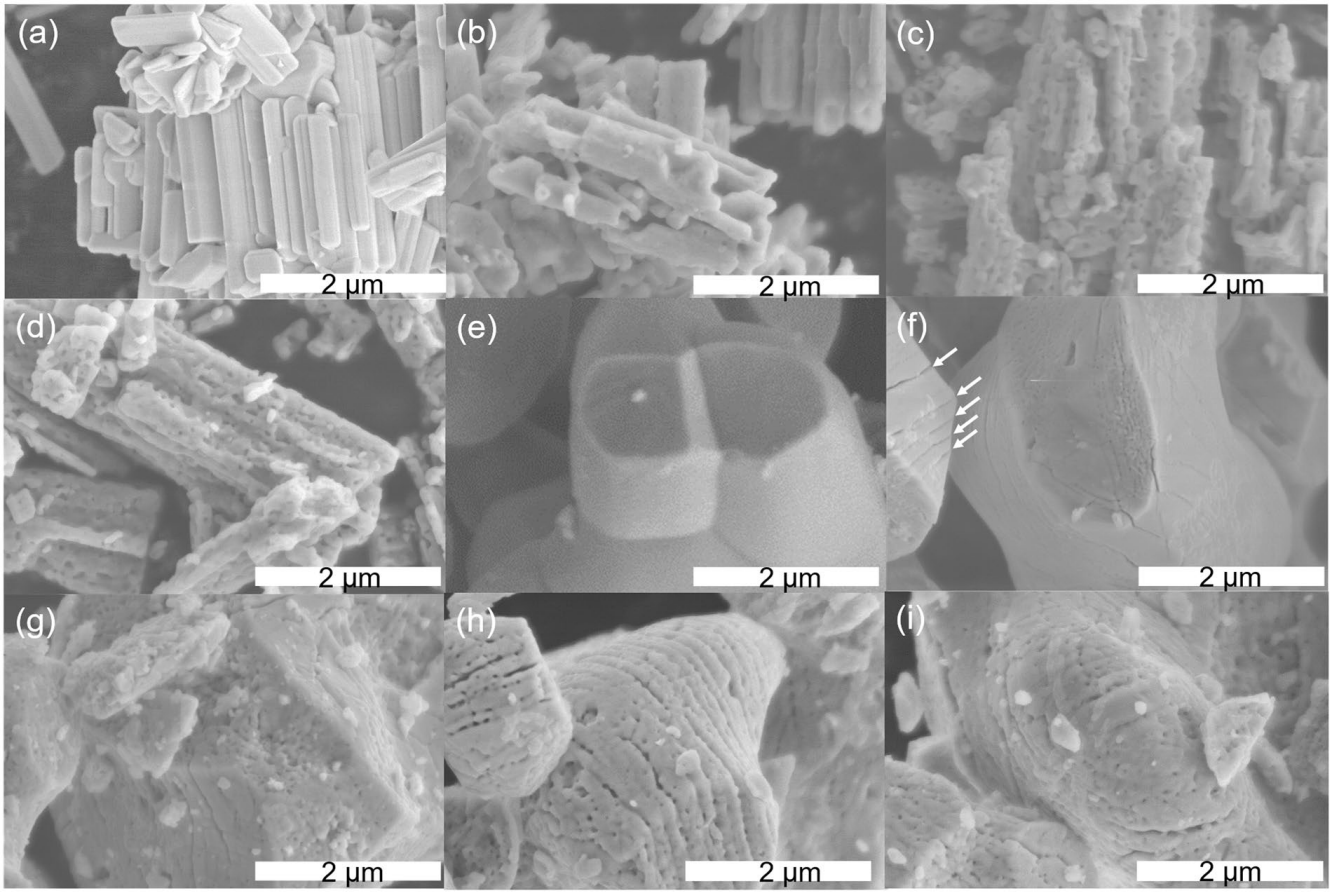
SEM images of (**a**) original Sr_2_Nb_2_O_7_, and products obtained after nitridation for (**b**) 1, (**c**) 5, and (**d**) 20 h, (**e**) original SrNbO_3_, and products obtained after nitridation for (**f**) 1, (**g**) 5, (**h**) 20 h, and (**i**) 40 h. The white arrows in (**f**) indicate parallel cracks.

The SEM images shown in Fig. 3 reveal that the Sr_2_Nb_2_O_7_ particles were composed of bundles of columnar crystals. The Sr_2_Nb_2_O_7_ particles were typically several micrometers in size but some were also much larger or smaller as shown in Fig. S2. The crystals gradually became more porous as the nitridation time was prolonged (See Fig. 3(b–d)). This trend has also been observed for (oxy)nitrides such as TaON, Ta_3_N_5_, and LaTiO_2_N^18–20^. In most instances of nitridation of oxides to form (oxy)nitrides, a lattice shrinkage occurs. In the case of nitridation of the layered perovskite-type compound Sr_2_Nb_2_O_7_, 2 mol of nitrogen is introduced while 3 mol of oxygen is extracted, resulting in a structural change from a layered perovskite to a perovskite. Since a layered perovskite structure has a lower density than a cubic perovskite, the lattice will shrink. Based on the observation that the secondary particles retained their original sizes after nitridation, it is most likely that porous oxynitrides were obtained after nitridation due to a lattice shrinkage. This porous structure is also supported by the nitrogen adsorption analysis data shown in Table S3, which indicates that the surface area was doubled following nitridation.

In the case of SrNbO_3_, large particles with a size of more than 5 μm were observed, and the average particle size was larger than that for Sr_2_Nb_2_O_7_. As shown in Table S3, the surface area for SrNbO_3_ was much less than that for Sr_2_Nb_2_O_7_ due to the presence of smoother surfaces and larger particles. One distinct difference from Sr_2_Nb_2_O_7_ particles was observed in the sample nitrided for 1 h. The SEM image in Fig. 3(f) demonstrates that parallel cracks were present in the sample nitrided for 1 h, probably suggesting the formation of Sr_5_Nb_4_O_15_ with a layered perovskite related structure. As the nitridation time increased, the surface morphology became porous and cracked, resulting in a steady increase in surface area, as shown in Table S3. In contrast to Sr_2_Nb_2_O_7_, nitridation from SrNbO_3_ did not involve a change in the crystal structure and so the crystals would be expected to expand as nitrogen was introduced. This effect is believed to have expanded the particles, leading to the formation of cracks and a phase separation.

**Figure 4:**
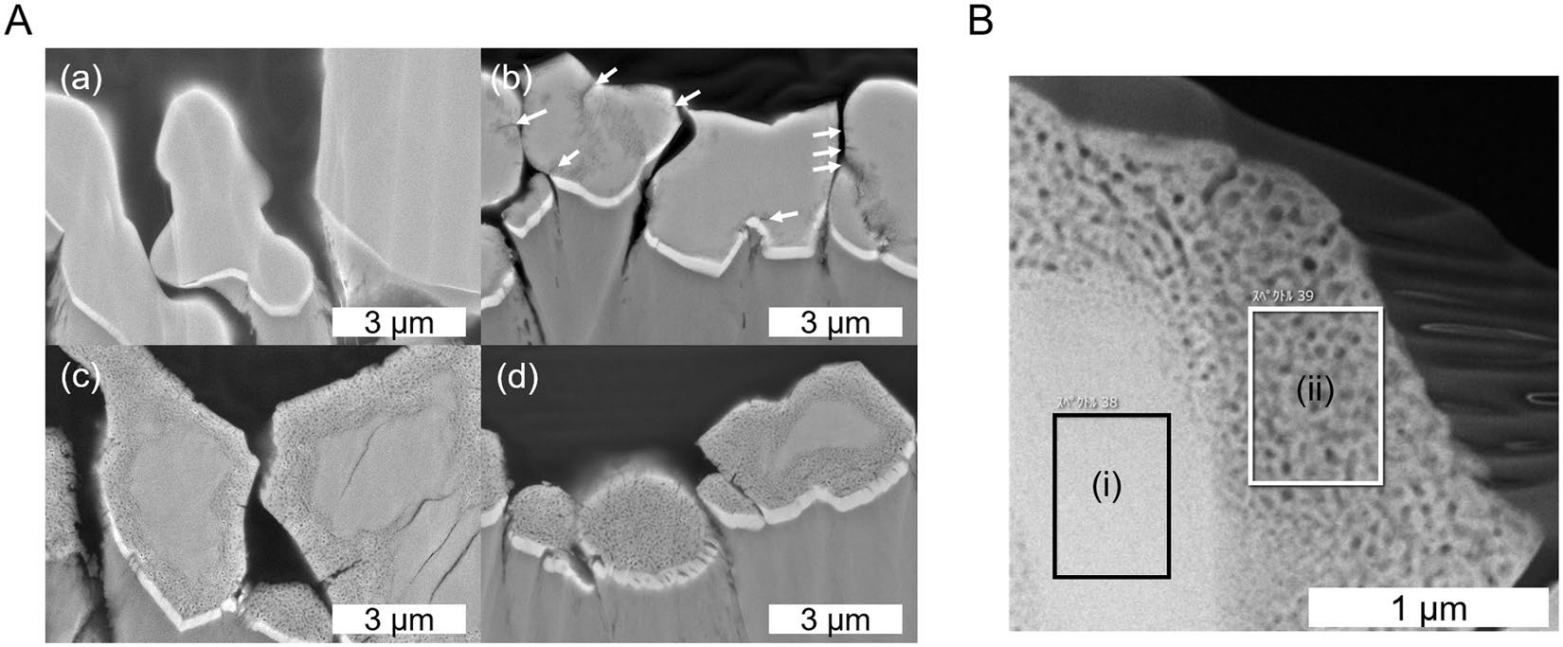
(**A**) Cross sectional SEM images of (a) SrNbO_3_/Nb/Ti and subsequently nitrided SrNbO_2_N/Nb/Ti nitrided for (b) 1 h, (c) 20 h and (d) 40 h. The white arrows in (b) indicate cracks. (**B**) STEM-DF image of SrNbO_2_N nitrided from SrNbO_3_ for 20 h. The N/O ratio by EDS in area (i) and (ii) is 0.28 and 0.41, respectively.

**Figure 5:**
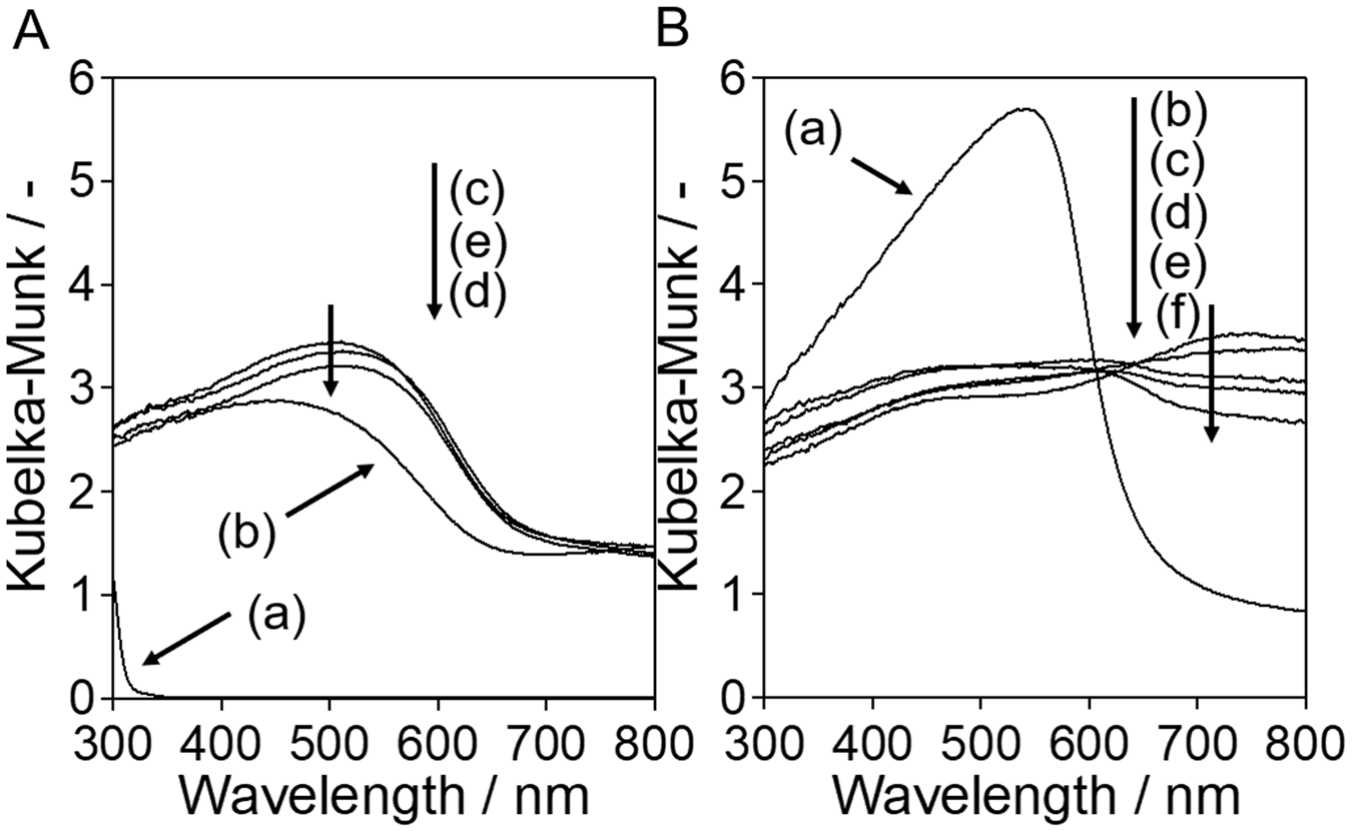
Diffuse reflectance spectra for SrNbO_2_N obtained by nitridation of (**A**) Sr_2_Nb_2_O_7_ and (**B**) SrNbO_3_. Legend: (a) oxide precursor and (b–f) samples obtained after nitridation times of (b) 1, (c) 5, (d) 10, (e) 20, and (f) 30 h.

Figure 4A shows the cross-sectional SEM images of SrNbO_2_N nitrided from SrNbO_3_ with various nitridation time. In this measurement, SrNbO_2_N/Nb/Ti photoelectrodes prepared by a particle transfer method were utilized for suppressing charge up during SEM observation. After nitridation for 1 h, some cracks and pores near surface appeared. In the case of nitridation for 20 h, there are obviously two regions like a “core-shell” structure, in which a “core” has smaller pores while a “shell” has larger pores. Even after nitridation for 40 h, some particles remained a “core-shell” structure while others completely became porous. The cross-sectional TEM image more precisely presented porous structure as shown in Fig. 4B. It was revealed that even “core” was porous although the pores were smaller than pores in “shell.” Although EDS analysis shows both “core” and “shell” contain certain amount of nitrogen, it is notable that the “shell” in Fig. 5 has larger N/O ratio of 0.41 than 0.28 in the “core,” indicating that the “core” was less nitrided.

### Optical properties

Figure 5 shows the diffuse reflectance (DR) spectra of the oxide precursors and the SrNbO_2_N samples obtained at different nitridation times. The original Sr_2_Nb_2_O_7_ particles had a white color and were only able to absorb light up to 320 nm. However, the absorption edge clearly shifted to longer wavelengths as nitridation proceeded. After nitridation for 1 h, the absorption edge was over 600 nm and reached 690 nm after 5 h. Further nitridation did not affect the optical properties. Absorption beyond the absorption edge as observed here is usually attributed to the formation of reduced Nb species or anion defects such as anion vacancies or O_N_ anti-site defects.

SrNbO_3_ exhibited strong absorption up to 650 nm and had a blood red coloration, both of which are consistent with previous results^11,12^. DR spectra of samples nitrided for less than 10 h did not show clear absorption edges and contained intense background signals over 700 nm. After nitridation for 20 h, an absorption edge appeared at 690 nm, although this edge was not well defined. It is notable that the SrNbO_3_ particles showed relatively minimal absorption beyond the absorption edge region in spite of the reduced valency of the Nb in this material, indicating that reduced Nb species were not responsible for the absorption above 700 nm. Therefore, it is probable that this absorption can be primarily attributed to anion defects. Moreover, the absorption above 700 nm decreased with increasing nitridation time more than 1 h, indicating that the number of anion defects first increased and then gradually decreased with progression of the nitridation.

### Relative weight analysis

**Figure 6:**
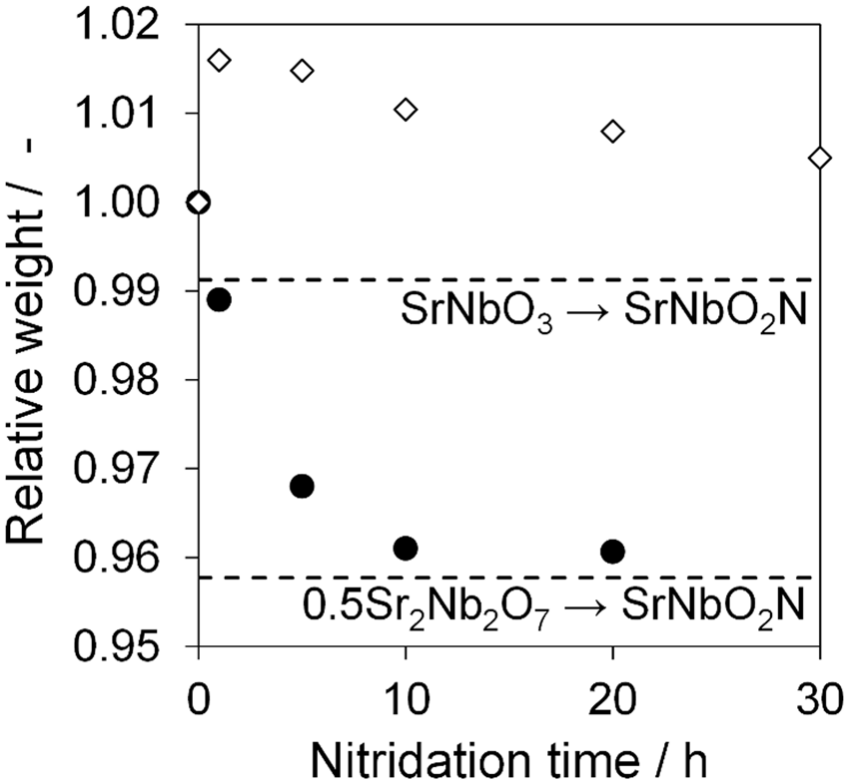
Relative weight of oxide precursors and nitrided samples as a function of nitridation time. Closed circles (●) and open diamonds (◇) indicate SrNbO_2_N synthesized from Sr_2_Nb_2_O_7_ and SrNbO_3_, respectively. Dashed lines indicate the expected value following complete nitridation.

Nitridation of the oxides to the oxynitride was further examined by following trends in the relative weight of the nitrided particles compared to the oxide precursors, using the equation provided in the Experimental section. This method can readily track the extent of nitridation. In the case of the Sr_2_Nb_2_O_7_ series, it was expected that the relative weight would decrease as a function of the nitridation time, reaching a value of 0.958 at complete nitridation. As shown in Fig. 6, the relative weight rapidly decreased over the initial 5 h, then further decreased up to 10 h, resulting in a plateau value close to the expected value. Thus, these relative weight data provide clear evidence for the progression of the expected nitridation. A similar trend has also been reported for the nitridation of La_2_Ti_2_O_7_-based oxides^21^. The elemental analysis data in Table S2 also support the above discussions. These values show that the O/Nb ratio steadily decreased while the N/Nb ratio increased, indicating that the introduction of nitrogen and the extraction of oxygen occurred simultaneously based on charge compensation.

Interestingly, a completely different trend was observed in the case of the SrNbO_3_ series. SrNbO_2_N has a formula weight (226.53 g mol^−1^) slightly less than that of SrNbO_3_ (228.53 g mol^−1^), and so it was anticipated that a similar trend to Sr_2_Nb_2_O_7_ series in the relative weight would be observed, with a gradual decrease in parallel with the anion exchange. However, as shown in Fig. 6, the relative weight initially increased, followed by a slow decrease. The elemental analysis data in Table S2 also show that the N/Nb ratio increased whereas the O/Nb ratio did not change from that in the oxide precursor after nitridation for 1 h, indicative of an increase in the total anion amount. One unique characteristic of SrNbO_3_ is that the Nb^4+^ must be oxidized to Nb^5+^ to form SrNbO_2_N during nitridation. In order to compensate for this, the Nb species must be oxidized during this process. Therefore, it appears that nitrogen was introduced during the initial stage of nitridation while oxygen ions remained in the crystals, followed by the subsequent extraction of the oxygen. After nitridation for 1 h, the relative weight became 1.016. When assumed that the increase of relative weight is only due to introduction of N species, the expected composition formula is SrNbO_3_N_0.26_. The XRD analysis as shown in Fig. 1 presents that Sr_5_Nb_4_O_15_ has formed at this stage. Therefore, it is more likely that the obtained product was a mixture of Sr_5_Nb_4_O_15-*x*_N_*x*_ and Sr_1-*y*_NbO_3-*z*_N_*z*_. To simplify this, considering the mixture is Sr_5_Nb_4_O_15_ and Sr_1-*x*_NbO_3-*y*_N_*y*_, it is roughly estimated that 30–40% of SrNbO_3_ has changed into Sr_5_Nb_4_O_15_.

Even after nitridation for 30 h, the relative weight did not reach the expected value, indicating that the nitridation of SrNbO_3_ did not proceed to completion. One possible reason is the larger particle size for the SrNbO_3_ oxide precursor, although the different crystal structures of SrNbO_3_ and Sr_2_Nb_2_O_7_ might also be responsible.

The effect of temperature during the nitridation of SrNbO_3_ was also investigated. As shown in Fig. S3, even at 1123 and 1223 K, similar trends were observed in the relative weight data. The application of higher temperatures during nitridation seems to accelerate the process, although the effect was limited.

### Photoelectrochemical performance

**Figure 7:**
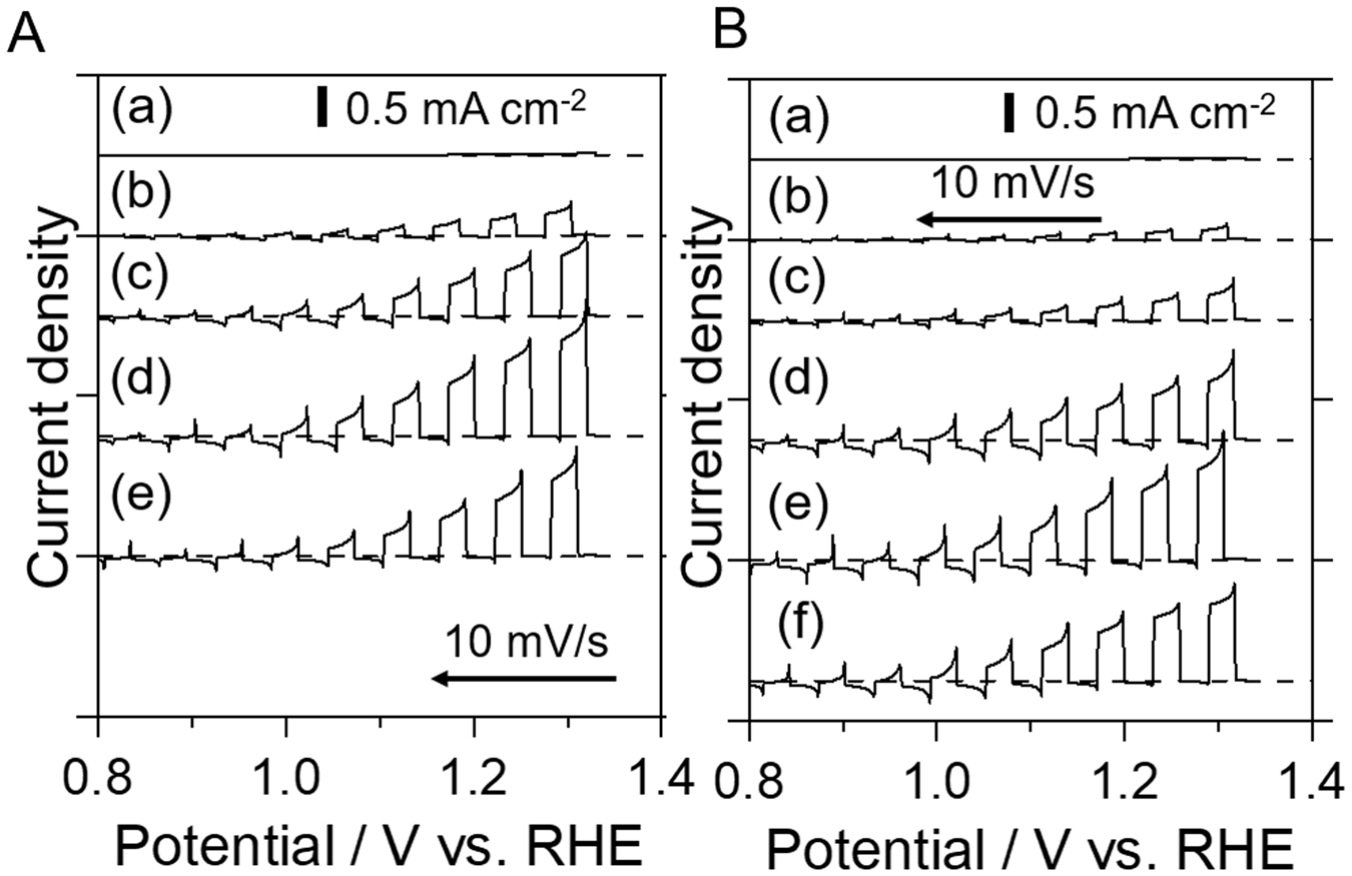
Current-potential curves for CoPi/SrNbO_2_N/Nb/Ti photoelectrodes synthesized from (**A**) Sr_2_Nb_2_O_7_ and (**B**) SrNbO_3_. Legend: (a) oxides, and samples nitrided for (b) 1, (c) 5, (d) 10, (e) 20, and (f) 30 h. Data were acquired under intermittent simulated sunlight (AM 1.5G) in an aqueous 0.2 M Na_3_PO_4_ solution adjusted to pH 13 by NaOH addition. Potentials were swept from positive to negative at a scan rate of 10 mV s^−1^.

PEC water splitting was performed using both the oxides and the oxynitrides. Figure 7A shows current-potential curves obtained from photoanodes made of Sr_2_Nb_2_O_7_ and the nitrided product SrNbO_2_N, modified using a CoPi cocatalyst. Under simulated sunlight, Sr_2_Nb_2_O_7_ did not show a clear photoresponse because of the minimal photon flux in the UV region. However, the photocurrent at a positive potential increased as the nitridation time was prolonged up to 10 h, reaching 0.77 mA cm^−2^ at 1.2 V vs. RHE, and then dropping after 20 h. The onset potential did not exhibit any clear dependence on the nitridation time. Moreover, since it was confirmed by the relative weight analysis that a nitridation time of 10 h was sufficient for Sr_2_Nb_2_O_7_, it is likely that the photocurrent reached maximum after completion of crystal structural change and that excess nitridation led to a degradation of the surface of the SrNbO_2_N. It is thus found that the PEC properties were relevant to the extent of nitridation proceeded.

As shown in Fig. 7B, an almost negligible photocurrent was observed when using CoPi/SrNbO_3_/Nb/Ti despite the SrNbO_3_ was able to absorb light up to 650 nm. This is consistent with our previous results^12^. A definite photoanodic current was generated even after nitridation for only 1 h. As the formation of SrNbO_2_N proceeded, the photocurrents steadily increased to reach a maximum of 0.75 mA cm^−2^ at 1.2 V vs. RHE after nitridation for 20 h, which was the comparable photocurrent to one obtained in Sr_2_Nb_2_O_7_ series. This value is almost twice that previously reported for SrNbO_2_N synthesized from SrNbO_3_. It is consistent that the optimum nitridation time was longer than that of Sr_2_Nb_2_O_7_ due to the slow nitridation process and a larger particle size. It is worth noting that SrNbO_2_N made from SrNbO_3_ was less crystalline and had a larger concentration of defects compared to the material prepared from Sr_2_Nb_2_O_7_ but exhibited a similar photocurrent.

### Proposed nitridation processes

Based on obtained results, we propose the nitridation processes of Sr_2_Nb_2_O_7_ and SrNbO_3_ as follows. In both cases, the nitridation processes via oxygen vacancy were considered. When assumed that active nitrogen species are NH_2_, for example, the nitridation process of Sr_2_Nb_2_O_7_ could be explained with equations as:

4 $${{\rm{O}}}_{{\rm{O}}}+{{\rm{H}}}_{2}\to {{{\rm{V}}}_{{\rm{O}}}}^{\cdot \cdot }+2{\rm{e}}^{\prime} +{{\rm{H}}}_{2}{\rm{O}}$$

5 $${{\rm{NH}}}_{2}+3{\rm{e}}^{\prime} +{{{\rm{V}}}_{{\rm{O}}}}^{\cdot \cdot }\to {{\rm{N}}}_{{\rm{O}}}^{\prime} +\,{{\rm{H}}}_{2}$$

6 $$2{{\rm{N}}}_{{\rm{O}}}^{\prime} +{{{\rm{V}}}_{{\rm{O}}}}^{\cdot \cdot }\to 2{{\rm{N}}}_{{\rm{N}}}$$

In the first step, oxygen atoms desorb and oxygen vacancies (V_O_) form (Eq. (4)). Next, nitrogen atoms are introduced to V_O_ and nitrogen anti sites (N_O_) are formed (Eq. (5)). Finally, oxygen vacancies disappear by changing the crystal structure (Eq. (6)). In total, 3 mol of oxygen atoms are exchanged by 2 mol of nitrogen. Desorbed O_2_ reacts with H_2_ to produce H_2_O. It is notable that overall nitridation process itself is not an oxidation-reduction reaction. Although the authors did not fully understand the actual active species of nitrogen, above equations indicate that the formation of anion vacancies is inevitable when nitridation process includes a crystal structural change.

**Figure 8:**
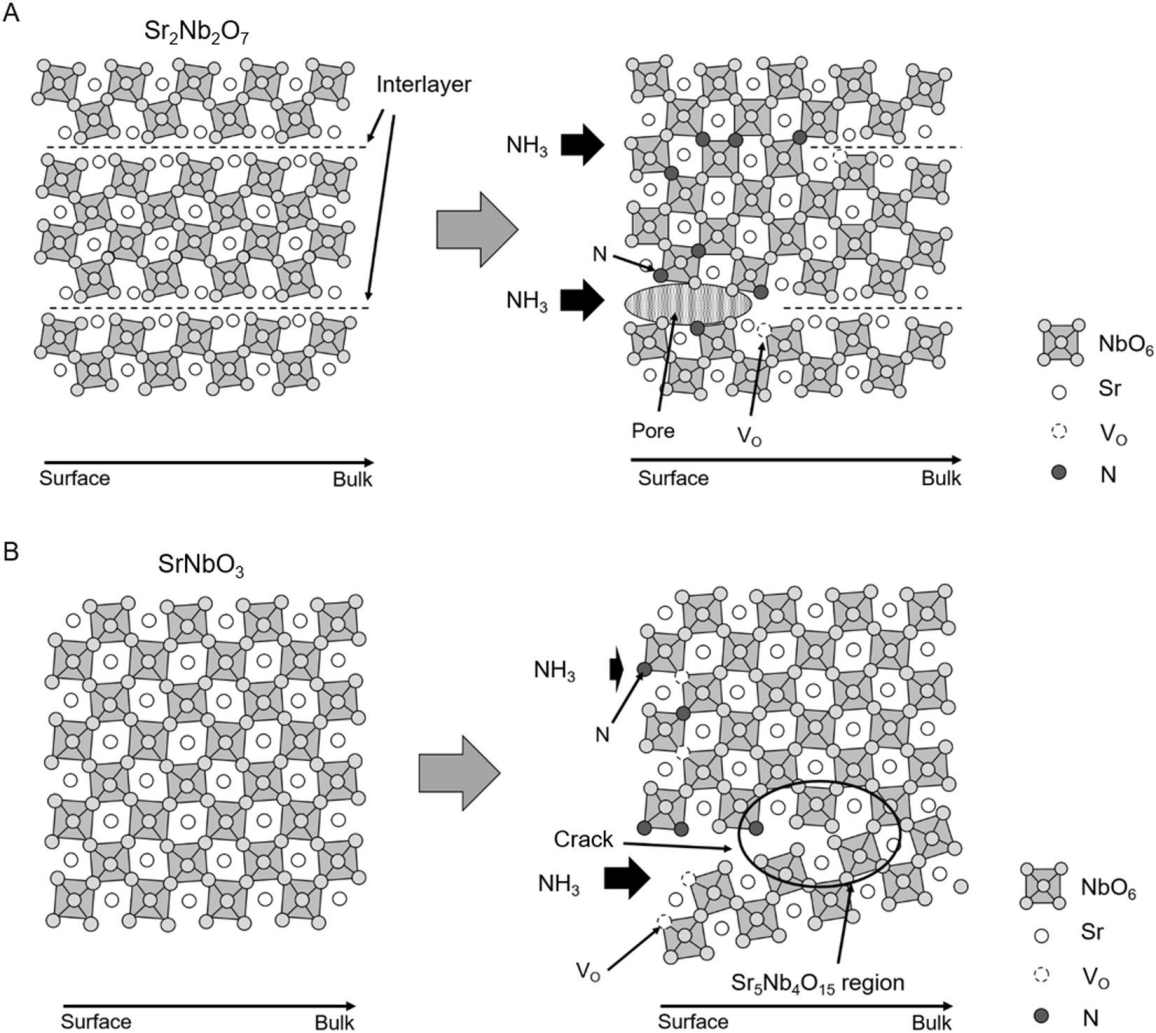
Schematic images of nitridation process of (Left) before and (Right) during nitridation of (**A**) a layered perovskite Sr_2_Nb_2_O_7_ and (**B**) a perovskite SrNbO_3_.

As shown in Fig. 8A, the layered perovskite-type material Sr_2_Nb_2_O_7_ has interlayers between the NbO_6_ layers for nitrogen to penetrate into the crystal. It has been reported that, in the case of an A_2_B_2_O_7_ oxide, nitrogen is introduced through spaces among layers by a so-called ‘zipper’ like mechanism, in which introduced nitrogen connects the interlayers to transform to the perovskite type structure^22^. Therefore, nitrogen could be exchanged with oxygen concurrently. Moreover, this anion exchange causes a lattice shrinkage. Considering the apparent particle size does not change during nitridation, it is most likely that this process leads the porous structures.

Contrary, in the case of nitridation of SrNbO_3_, Eq. (6) should be exchanged by the following equation:

6&x02032; $${{\rm{N}}}_{{\rm{O}}}^{\prime} \to {{\rm{N}}}_{{\rm{N}}}+{\rm{e}}^{\prime} $$

This process does not contain any crystal structural changes. Furthermore, this total nitridation process is an oxidation reaction. As a result, reduced Nb^4+^ should be oxidized to Nb^5+^ after nitridation. When step (5) or (6′) are slow, oxygen vacancies stimulate in the crystals. It is reasonable that the oxidation of Nb^4+^ was challenging under a reductive atmosphere during nitridation. Therefore, it is most likely that considerable number of oxygen vacancies remain in SrNbO_3_ and obtained products, leading to the large absorption over 700 nm (Fig. 5B(b–f)). Since introduction of oxygen vacancies and nitrogen species both expand the lattice constant (the lattice mismatch of oxide and resulting oxynitride is 0.7%), this causes the crack formation (Fig. 3(f)) to solve the stimulated distortion as shown in Fig. 8B. Our group has reported that *A* site poor perovskite Ba_1-*x*_NbO_3-δ_ was nitrided with maintaining the perovskite type structure^12^. In this case, the lattice mismatch reduced to only 0.1% at Ba_0.84_NbO_3-δ_. Although 0.7% of the lattice mismatch in SrNbO_3_ system is relatively small in terms of solid solution formation, it was seen that the small lattice mismatch played the critical role in this nitridation process. Formation of cracks requires the increase of the total amount of anion species, resulting in a phase separation of Sr_5_Nb_4_O_15_ and an increase of the relative weight at the initial stage of nitridation (Fig. 6). Additionally, some Nb^4+^ species are supposed to be oxidized to Nb^5+^ in this step. As nitridation proceeded, both SrNbO_3_ and Sr_5_Nb_4_O_15_ are nitrided to the final product, SrNbO_2_N.

Judging from the lattice constants, in the nitridation of SrNbO_3_ to SrNbO_2_N, a lattice shrinkage does not seem to proceed. It is thus likely that the porous structure was inevitably obtained due to exchanging oxygen and nitrogen species regardless of the extent of a lattice mismatch. In contrast to Sr_2_Nb_2_O_7_ oxides, a perovskite-type oxide SrNbO_3_ does not have interlayers to accelerate the diffusion of nitrogen sources. Therefore, it is more likely that the diffusion of nitrogen is much slower in this material than in Sr_2_Nb_2_O_7_. Although the formation of Sr_5_Nb_4_O_15_ partly contribute to the anion diffusion, SrNbO_3_ requires more severe conditions, such as the longer nitridation time and higher nitridation temperature than Sr_2_Nb_2_O_7_. These differences in the nitridation process are primarily the result of variations in the crystal structure and the valency of the cations in the oxide precursors. The resulting photocurrents were also affected by the nitridation processes. Photocurrent reached maximum after 10 h in the case of Sr_2_Nb_2_O_7_ series while 20 h of nitridation was optimum for SrNbO_3_ series. In the case of nitridation of SrNbO_3_, nitridation speed is dominated by a diffusion of nitrogen species. Therefore, particle size should play a critical role in nitridation time and resulting PEC properties. Since, in this paper, SrNbO_3_ was synthesized at relatively high temperature, 1773 K, it was challenging to reduce a particle size. Further development of synthesis procedure to control a particle size should improve PEC properties. Moreover, in both nitridation processes, anion vacancies inevitably formed, and this probably lead the limited photocurrent and positive onset potentials. It is therefore a key to develop a nitridation process to reduce the anion vacancies for realizing the solar hydrogen production using oxynitride materials.

## Conclusion

In conclusion, the nitridation processes for two oxide precursors, the layered perovskite-type Sr_2_Nb_2_O_7_ and the perovskite-type SrNbO_3_, to produce the perovskite-type material SrNbO_2_N were examined. The relationship between the PEC properties of the products and the initial oxide were also investigated. Nitridation without any change in crystal structure was partly achieved in nitridation of SrNbO_3_, although formation of an impurity phase was detected. The data also suggest that nitridation from SrNbO_3_ proceeds via a different pathway from Sr_2_Nb_2_O_7_, with variations in lattice expansion and oxidation of Nb cations. Nitridation models based on the crystal structure were presented, in which nitridation processes triggered by formation of oxygen vacancies were considered. The photocurrent obtained from the products was correlated with the extent of nitridation determined from the relative weight analysis. This study demonstrates the importance of understanding the nitridation mechanism for the oxide precursor, focusing on the crystal structure. The knowledge obtained in this study should be widely applicable to other oxynitrides.

## Electronic supplementary material

Supplementary information

## References

[1] Fujishima A, Honda K (1972). Electrochemical photolysis of water at a semiconductor electrode. Nature.

[2] Kudo A, Miseki Y (2009). Heterogeneous photocatalyst materials for water splitting. Chem. Soc. Rev..

[3] Higashi M, Domen K, Abe R (2011). Fabrication of efficient TaON and Ta3N5 photoanodes for water splitting under visible light irradiation. Energy Environ. Sci..

[4] Liu G (2016). Enabling an integrated tantalum nitride photoanode to approach the theoretical photocurrent limit for solar water splitting. Energy Environ. Sci..

[5] Pan C (2015). A complex perovskite-type oxynitride: The first photocatalyst for water splitting operable at up to 600 nm. *Angew*. Chemie - Int. Ed..

[6] Ueda K (2015). Photoelectrochemical oxidation of water using BaTaO2N photoanodes prepared by particle transfer method. J. Am. Chem. Soc..

[7] Hisatomi T (2013). Photocatalytic oxygen evolution using BaNbO2N modified with cobalt oxide under photoexcitation up to 740 nm. Energy Environ. Sci..

[8] Oehler F, Ebbinghaus SG (2016). Photocatalytic properties of CoO x -loaded nano-crystalline perovskite oxynitrides ABO2N. Solid State Sci..

[9] Kim Y, Woodward PM, Baba-kishi KZ, Tai CW (2004). Characterization of the Structural, Optical, and Dielectric Properties of Oxynitride Perovskites AMO 2 N (A) Ba, Sr, Ca; M) Ta, Nb. Chem. Mater..

[10] Kodera M (2016). Effects of flux synthesis on SrNbO_2_N particles for photoelectrochemical water splitting. J. Mater. Chem. A.

[11] Xu X, Randorn C, Efstathiou P, Irvine JTS (2012). A red metallic oxide photocatalyst. Nat. Mater..

[12] Seo J (2016). Photoelectrochemical Water Splitting on Particulate ANbO2N (A = Ba, Sr) Photoanodes Prepared from Perovskite-Type ANbO3. Chem. Mater..

[13] Hellwig A, Hendry A (1994). Formation of barium-tantalum oxynitrides. J. Mater. Sci..

[14] Brophy MR, Pilgrim SM, Schulze WA (2011). Synthesis of BaTaO2N powders utilizing NH3 decomposition. J. Am. Ceram. Soc..

[15] Istomin SYa, Svensson G, yachenko OGD, Holm W, Antipov EV (1998). A Synthesis, Structure, and Electron Microscopy Study. J. Solid State Chem..

[16] Minegishi T, Nishimura N, Kubota J, Domen K (2013). Photoelectrochemical properties of LaTiO_2_N electrodes prepared by particle transfer for sunlight-driven water splitting. Chem. Sci..

[17] Kanan MW, Nocera DG (2008). *In Situ* Formation of an Water Containing Phosphate and Co_2_+. Science..

[18] Lu D (2004). Porous Single-Crystalline TaON and Ta3N5 Particles. Chem. Mater..

[19] Pokrant S, Cheynet MC, Irsen S, Maegli AE, Erni R (2014). Mesoporosity in photocatalytically active oxynitride single crystals. J. Phys. Chem. C.

[20] Pokrant S, Dilger S, Landsmann S (2016). Morphology and mesopores in photoelectrochemically active LaTiO_2_N single crystals. J. Mater. Res..

[21] Ebbinghaus SG (2009). Perovskite-related oxynitrides - Recent developments in synthesis, characterisation and investigations of physical properties. Prog. Solid State Chem..

[22] Ebbinghaus SG, Aguiar R, Weidenkaff A, Gsell S, Reller A (2008). Topotactical growth of thick perovskite oxynitride layers by nitridation of single crystalline oxides. Solid State Sci..

