# Supplementary material for: Investigation on nitridation processes of Sr2Nb2O7 and SrNbO3 to SrNbO2N for photoelectrochemical water splitting
Source: Sci Rep. 2018 Oct 26;8:15849. doi: 10.1038/s41598-018-34184-2 (PMC6203854; doi:10.1038/s41598-018-34184-2)
Supplement: Supplementary file 1 — Supplementary information [file 41598_2018_34184_MOESM1_ESM.pdf]

Supporting Information

Investigation on nitridation processes of $Sr_2Nb_2O_7$ and $SrNbO_3$ to $SrNbO_2N$ for photoelectrochemical water splitting

Masanori Kodera[1], Yosuke Moriya[1, †], Masao Katayama[1], Takashi Hisatomi[1], Tsutomu Minegishi[1], and Kazunari Domen[1,2]*

1 Department of Chemical System Engineering, School of Engineering, The University of Tokyo, 7-3-1 Hongo, Bunkyo-ku, Tokyo 113-8656, Japan

2 Center for Energy & Environmental Science, Shinshu University,
4-17-1 Wakasato, Nagano 380-8553, Japan

† Present address: Global Aqua Innovation Center for Improving Living Standards and Water-sustainability, Shinshu University, 4-17-1 Wakasato, Nagano 380-8553, Japan

* *Corresponding author*

Contents

Table S1 FWHM values for the (110) diffraction peaks of the initial perovskite-type oxides and products following various nitridation times.

Table S2 Elemental analysis data for $SrNbO_2N$ specimens obtained by nitriding (A) $Sr_2Nb_2O_7$ and (B) $SrNbO_3$.

Table S3 BET surface areas of oxide precursors and $SrNbO_2N$ specimens obtained from these precursors.

Fig. S1 XRD patterns for $SrNbO_2N$ obtained from nitridation of the $SrNbO_3$ oxide precursor.

Fig. S2 Low-magnification SEM images of (a) $Sr_2Nb_2O_7$ and (b) $SrNbO_3$.

Fig. S3 Relative weight for $SrNbO_2N$ obtained by nitridation of $SrNbO_3$ at 1123 and 1223 K as function of nitridation time.

Table S1 FWHM values for (110) diffraction peaks for initial perovskite-type oxides and products following various nitridation times.

Nit. time / h	FWHM of (110) / degree	
	From $Sr_2Nb_2O_7$	From $SrNbO_3$
0	-	0.10
1	-	-
5	0.25	0.24
10	0.24	0.32
20	0.24	0.27
30	-	0.27

Table S2 Elemental analysis data for $SrNbO_2N$ specimens obtained by nitriding (A) $Sr_2Nb_2O_7$ and (B) $SrNbO_3$. Ideal stoichiometric values for $Sr_2Nb_2O_7$, $SrNbO_3$, and $SrNbO_2N$ are also presented. Nitridation time of 0 h indicates oxide precursors before nitridation.

A

Nit. time	Sr/Nb*	Nb/Nb*	O/Nb**	N/Nb**
$Sr_2Nb_2O_7$	1	1	3.5	0
0	1.00	1.00	3.31	0.00
1	0.97	1.00	2.58	0.48
5	0.98	1.00	2.09	1.05
10	0.97	1.00	2.02	1.06
20	0.95	1.00	1.95	1.04
$SrNbO_2N$	1	1	2	1

* by ICP-AES

** by Oxygen-Nitrogen analysis

Al < 0.1 wt%, Rb < 0.1 wt%

B

Nit. time	Sr/Nb*	Nb/Nb*	O/Nb**	N/Nb**
$SrNbO_3$	1	1	3	0
0	0.96	1.00	2.69	0.00
1	0.98	1.00	2.69	0.37
5	0.97	1.00	2.27	0.74
10	0.97	1.00	2.05	0.91
20	0.98	1.00	1.86	1.09
30	0.96	1.00	1.82	1.11
$SrNbO_2N$	1	1	2	1

* by ICP-AES

** by Oxygen-Nitrogen analysis

Table S3 BET surface areas of oxide precursors and $SrNbO_2N$ specimens obtained from these precursors.

Nitridation time / h	Surface area / m^2 g^{-1}	
	From $Sr_2Nb_2O_7$	From $SrNbO_3$
0	4.7	0.2
1	4.3	1.0
5	10.2	2.2
10	10.1	3.1
20	9.9	5.8
30	-	5.7

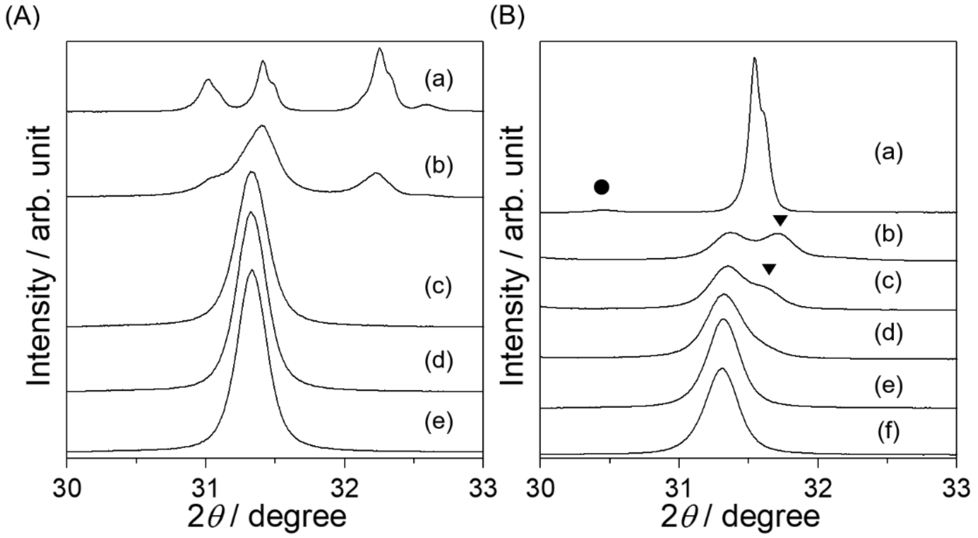

Fig. S1 XRD patterns for $SrNbO_2N$ obtained by nitridation of (A) $Sr_2Nb_2O_7$ and (B) $SrNbO_3$ oxide precursor. Legend: (a) oxide precursor and (b-f) nitrided samples obtained for nitridation times of (b) 1, (c) 5, (d) 10, (e) 20, and (f) 30 h. Closed triangles and a closed circle indicate $Sr_5Nb_4O_{15}$ and $Sr_7Nb_6O_{21}$, respectively.

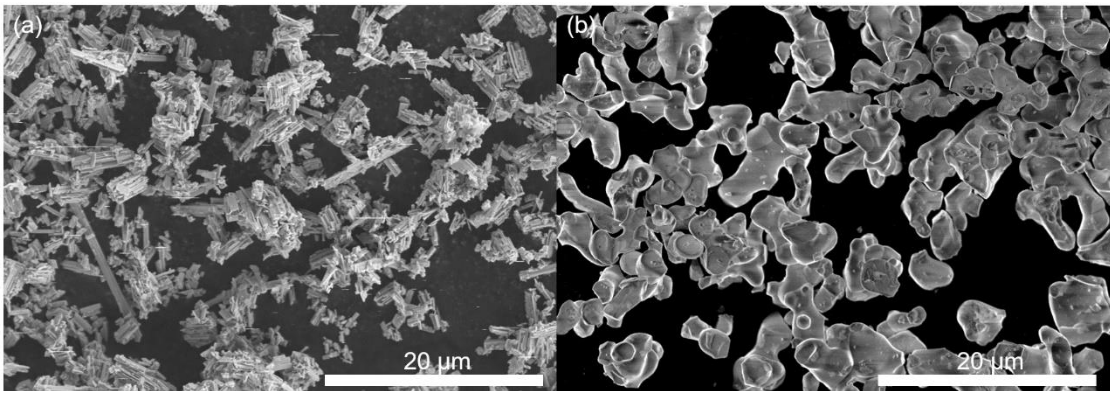

Fig. S2 Low-magnification SEM images of (a) $Sr_2Nb_2O_7$ and (b) $SrNbO_3$.

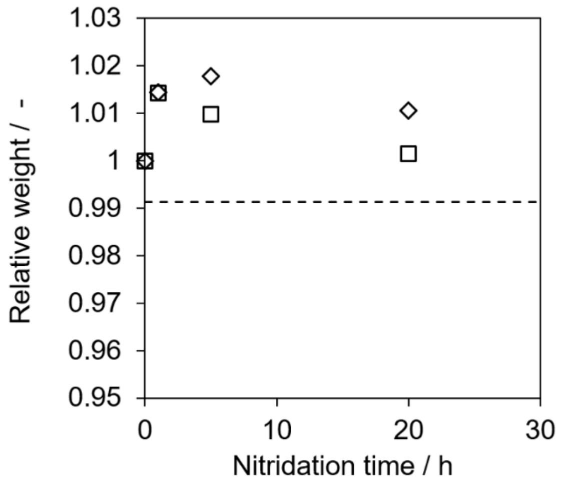

Fig. S3 Relative weight of oxide precursors and nitrided samples as a function of nitridation time. Open diamonds (◇) and open square (□) indicate $SrNbO_2N$ nitrided at 1123 K and 1223 K from $SrNbO_3$ oxide precursors, respectively. Dashed line indicates the expected value following complete nitridation.
